# Emerging Sensing Technologies for Liquid Biopsy Applications: Steps Closer to Personalized Medicine

**DOI:** 10.3390/s24247902

**Published:** 2024-12-11

**Authors:** Panagiota M. Kalligosfyri, Eleni Lamprou, Despina P. Kalogianni

**Affiliations:** Department of Chemistry, University of Patras, GR26504 Patras, Greece; eleni_lamprou@upatras.gr

**Keywords:** microRNA, circulating tumor DNA, circulating tumor cells, exosomes, optical sensors, lateral flow assay, colorimetric sensors, fluorescent sensors, SPR, SERS

## Abstract

Liquid biopsy is an efficient diagnostic/prognostic tool for tumor-derived component detection in peripheral circulation and other body fluids. The rapid assessment of liquid biopsy techniques facilitates early cancer diagnosis and prognosis. Early and precise detection of tumor biomarkers provides crucial information about the tumor that guides clinicians towards effective personalized medicine. Point-of-care-testing remains still a great challenge in cancer diagnostics. Liquid biopsy is a promising alternative to tissue biopsy with the great advantages of less invasion and real-time monitoring of the disease, also providing information about tumor heterogeneity. The field is continuously and rapidly expanding. Numerous sophisticated biosensors have been developed targeting several biomarkers to achieve low detection limits, increased specificity and robustness. Current biosensors include mainly optical sensors, such as colorimetric, fluorescent, SPR, SERS and lateral flow assays. Electrochemical sensors have also been developed, providing very low detection limits. Colorimetric sensors exhibited simplicity in signal interpretation, while fluorescent sensors contributed to low analysis times, and SPR/SERS enabled label-free and rapid analysis. Novel target amplification and signal enhancement techniques have been exploited to increase the detectability of the sensors. In this context, this review is focused on the recent advances in biosensing technology for cutting-edge liquid biopsy applications towards point-of-care testing.

## 1. Introduction

Liquid biopsy has gained widespread attention in recent years. Liquid biopsy is a minimally invasive method that analyzes bodily fluids like blood, serum, urine or sweat to detect and monitor disease-related biomarkers. It is characterized by strong advantages like real-time and multicomponent monitoring of any type of cancer, qualitative and quantitative analysis, early detection of the metastasis and long-term observation of the disease at the cellular, genetic and molecular levels. Frequent sampling may also occur. It is a very promising and emerging diagnostic technology alternative to tissue biopsy [1,2]. Tissue biopsy is a strongly invasive technique that may have various risks for the patient, but the most significant disadvantage is that it does not provide information about tumor heterogeneity [3,4]. Liquid biopsy, on the other hand, provides crucial information about the tumor. Liquid biopsy biomarkers include microRNAs, non-coding RNAs, circulating cell-free RNA, circulating tumor DNA (ctDNA), circulating tumor cells (CTCs), extracellular vesicles (EVs) and exosomes, proteins, tumor-associated antigens and other metabolites found in various body fluids [5,6]. Circulating nucleic acids are more abundant than CTCs; thus, their detection provides higher detectability than CTCs or tumors, while exosomes have higher stability than other biomarkers in body fluids [7]. However, there is still a lack of accurate early diagnostic methods for several types of cancer. Therefore, new biomarkers have to be investigated and applied for liquid biopsy [5,6]. Novel biomarkers that are not well studied yet are PIWI-interacting RNAs (piRNAs), which are non-coding RNAs that bind specifically to the PIWI subfamily of Argonaute proteins and are usually found in exosomes [8].

Traditional methods for biomolecular analysis are usually tedious and time-consuming, requiring highly trained users as well as expensive and complex instrumentation. On the other hand, biosensor development is aimed at enhancing simplicity and user-friendly formats, providing fast analysis, high detectability and specificity, portability, cost-effectiveness, versatility and multiplicity potential. Also, one major advantage of biosensing technology is the ease of result interpretation [3]. In this regard, artificial intelligence/machine learning and image processing tools have appeared recently in the foreground to enhance the easiness, accuracy, clinical sensitivity and specificity of the results. Smartphone-based detection systems have also enabled this approach [9,10]. There are several methods and biosensors for the detection of liquid biopsy biomarkers. The greatest challenge in liquid biopsy applications is the detection of extremely low concentrations of these biomarkers in body fluids, along with their instability. For these reasons, amplification techniques are required for sensitive detection [11].

**Figure 1:**
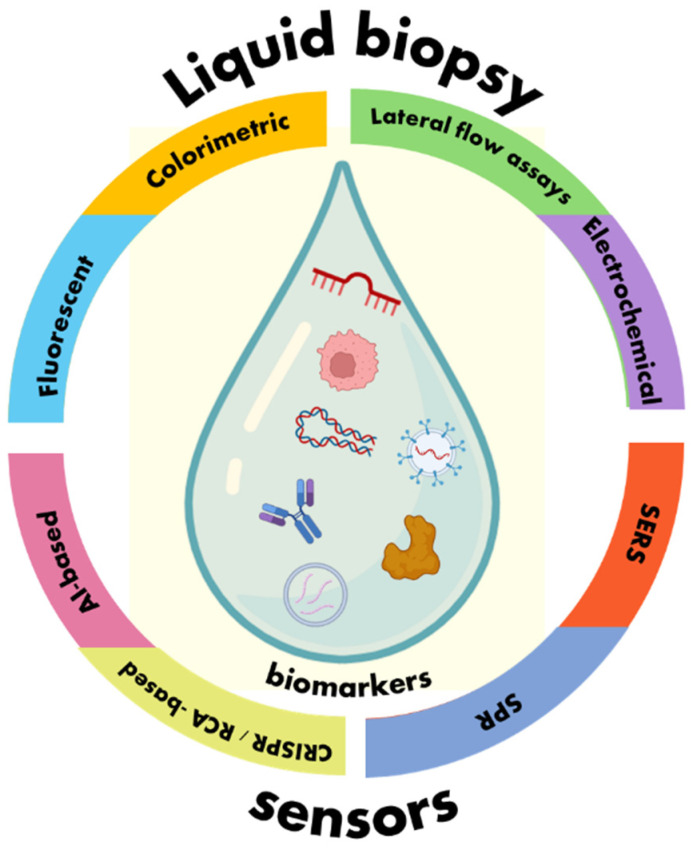
Overview of sensors for cutting-edge liquid biopsy applications.

Amplification techniques include target and/or signal amplification using various methods and nanomaterials and target recycling to increase signal outputs. Target amplification accounts for nucleic acids and includes reverse transcription polymerase chain reaction (RT-PCR), real-time PCR, digital PCR, exponential amplification reaction (EXPAR) and other isothermal amplification techniques such as Loop-Mediated Isothermal Amplification (LAMP), recombinase polymerase amplification (RPA) and rolling circle amplification (RCA). Target recycling and signal amplification methods have been achieved by hybridization chain reaction (HCR), RCA, Strand Displacement Reaction (SDR or SDA), catalytic hairpin assembly (CHA), duplex-specific nuclease (DSN), nanoenzymes such as DNAzymes, Nickzymes and Clustered Regularly Interspaced Short Palindromic Repeats (CRISPR). Many different nanomaterials and nanoparticles have been used to increase the signal, especially in surface plasmon resonance (SPR) and Surface-Enhanced Raman Scattering (SERS) spectroscopic techniques [2,12]. An emerging amplification technique is based on multicomponent nucleic acid enzymes (MNAzymes) that can be a more versatile and functional alternative to DNAzymes and consist of two partzymes: a part that binds partially to a substrate that is cleaved by the MNAzyme, and a sensing part that is hybridized to the target nucleic acid sequence. The MNAzyme is activated by the target and binds and cleaves many substrate molecules, leading to increased signal outputs [12,13]. As biosensing technology is rapidly expanding, also towards personalized medicine, this review is focused on the development of emerging sensors for the detection of various cancer-related biomarkers in body fluids. An overview of the performance of these sensors is provided, and future perspectives are also discussed. Signal amplification based on CRISPR-Cas proteins and RCA, as strongly adopted by researchers, is separately presented. The currently developed sensors for cutting-edge liquid biopsy applications are summarized in Figure 1.

Finally, in this comprehensive review, we aim to categorize sensors according to their detection principles and further classify them based on the biomarkers they target, with a particular focus on recent advancements and trends from the past year in liquid biopsy sensor technologies. Additionally, this review highlights emerging technologies, innovative strategies, novel materials and the enhanced capabilities of these sensors, while critically examining their limitations and proposing future directions for their development.

## 2. Colorimetric Sensors

Colorimetric-based methods and sensors exhibit the advantage of practicality and simplicity, as the readout is accomplished by the naked eye, a simple photometer or microplate reader, or a mobile device such as a smartphone, providing a low-cost analysis and enabling analysis in the field. Therefore, no sophisticated and expensive instrumentation is needed [14]. The rapid expansion of nanotechnology has increased the overall analytical performance of biosensors due to the use of novel nanomaterials with unique optical properties. Colored nanoparticles, such as gold nanoparticles, increased the specificity and detectability of biosensors due to their exceptional properties. Most current methods are focused on the selective detection of exosomes. New cancer biomarkers on the exosome surface have been targeted. Aptamers and novel nanoparticles, nanozymes and DNAzymes, have been introduced to produce enhanced visual signals, lower the cost and the analysis time and increase the specificity and detectability of the sensors. Sophisticated amplification strategies were still combined with the above materials for signal enhancement. Multiplex analysis was also achieved with colorimetric sensors.

### 2.1. MicroRNAs

In a current approach, gold nanoparticles (AuNPs) and multicomponent nucleic acid enzymes (MNAzymes) for signal enhancement have been exploited for the rapid spectroscopic/colorimetric detection and quantification of miR-4739 in blood samples related to breast cancer. An alternative strategy involved the activation of the MNAzyme from the miRNA target that catalyzed the cleavage of a substrate. The MNAzyme was released and activated to interact with other substrate molecules, leading to signal enhancement. DNA-functionalized AuNPs, complementary to the substrate at both ends, were used. In the presence of the substrate, the AuNPs were hybridized to the substrate, inducing the aggregation of the nanoparticles with subsequent color change. In the presence of the miRNA target, the substrate was cleaved upon MNAzyme activation, and the AuNPs were kept in a suspension. Finally, the free AuNPs in the solution were measured photometrically, providing a detection limit of 7 pM. The sensor had good selectivity among different miRNAs but revealed low specificity when a single-mismatched miRNA was used. Finally, the sensor could be applied for the detection of multiple miRNAs by simply changing the nanoparticles used with a different absorbance peak [12].

### 2.2. Circulating Tumor DNA (ctDNA)

A novel triple-circulating amplification system integrating entropy, enzyme cascade-driven 3D DNA walker and Branched Hybridization Strand Reaction (B-HCR) was developed for the detection of breast cancer-related ctDNA, in particular, a PIK3CA^E545K^ mutation. DNA walkers have emerged as an attractive amplification strategy with a higher DNA load that increases the efficiency of the interactions. In the study described, a DNA walker was triggered by the target, and a Strand Displacement Reaction (SDR) was initiated. The DNA walker contained a DNAzyme sequence that cleaved a substrate, producing triggers for a subsequent hybridization chain reaction (HCR) that resulted in the formation of a G-quadruplex structure. As a result of the addition of hemin, H_2_O_2_ and ABTS, color production was observed. The method had a detection limit of 0.65 fM with very good specificity [15].

### 2.3. Exosomes

**Figure 2:**
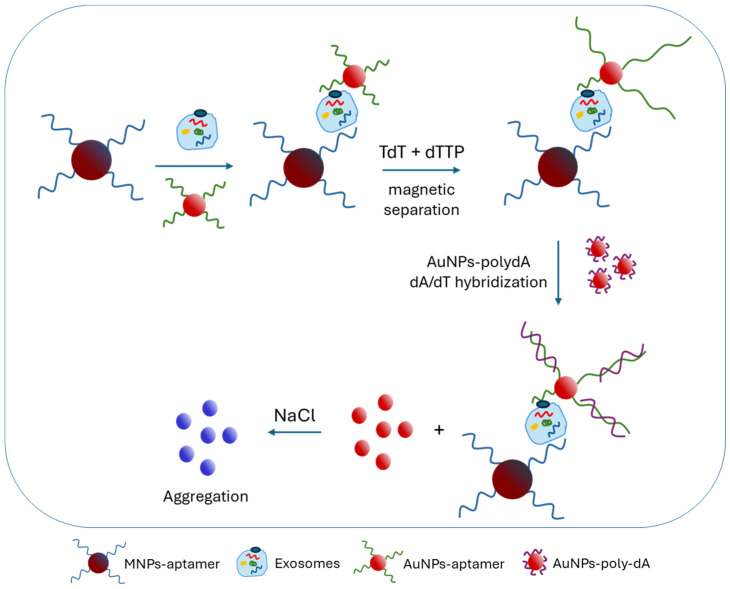
A colorimetric sensor for the detection of exosomes based on AuNP aggregation after aptamer-based capturing of exosomes, TdT elongation of the aptamers and NaCl addition [16].

A novel colorimetric method has been developed for exosome detection for POCT. Exosomes were first captured by aptamer–magnetic nanoparticle conjugates and isolated with a magnet. Then, aptamer/nucleolin-AuNPs were specifically bound to the exosomes, and the aptamers were afterward elongated with a polydT sequence by terminal deoxynucleotidyl transferase (TdT). Finally, polydA-AuNPs were added, resulting in the aggregation of AuNPs and a color change in the presence of NaCl through dA/dT hybridization (Figure 2). This system provided an LOD of 45 particles/μL and exhibited good analytical performance due to magnetic separation [16]. Another approach also used aptamers attached to a microplate on which exosomes were captured, as well as nucleolin aptamers attached to AuNPs which were also bound to the exosomes. AuNPs carried a DNA probe that triggered a subsequent HCR, generating multiple DNA fragments with G-quadruplex sequences that, upon the addition of hemin, catalyzed the oxidation of TMB in the presence of H_2_O_2_, producing a blue color visible by the naked eye. This system provided a low detection limit of 50 particles/μL as a dual amplification system was used and the potential for on-site testing [17]. An ArGO@PDA@CeO_2_/ATP nanozyme was constructed for exosome detection and consisted of polydopamine-functionalized reduced graphene oxide with absorbed CeO_2_ nanosheets (rGO@PDA@CeO_2_). The system exhibited peroxidase activity which was enhanced by ATP. The activity was monitored by a colorimetric substrate (TMB) and H_2_O_2_. The absorption of aptamers on the nanosheets hindered the ATP enhancement, whereas, in the presence of the target, the aptamers were attached specifically to the exosomes, and the peroxidase activity was increased. This assay achieved a detection limit of 3.8 × 10^5^ particles/mL within 25 min [14]. Moreover, novel polydiacetylene (PDA) vesicle-based aptasensors exhibited a color shift in response to biological recognition. Anti-EpCAMs (epithelial cell adhesion molecules) and aptamer-conjugated PDA were used for the fast detection of exosomes within 15 min with high specificity, by causing a color shift from blue to purple after binding to PDA. The detection limit was ~3.7 × 10^4^ particles/μL. The sensor was applied for successful on-site detection of samples, being promising for on-site cancer diagnosis [18].

## 3. Fluorescent Sensors

Fluorescent sensors were developed in an attempt to increase detectability. They exploited labeling with fluorescent dyes or fluorescent nanoparticles along with signal enhancement techniques such as HCR, RCA and others. Fluorescence energy transfer (FRET) using several nanomaterials has been also used to develop homogeneous assays and minimize the need for extra labels. For low-cost analysis, mobile devices have been integrated as detection systems. Intercalating fluorescent dyes have gained interest due to their low cost, fast analysis and label-free detection. Aptamers conjugated to fluorescent nanomaterials gave superior performance compared to antibodies, due to their ease of synthesis with low cost, low toxicity, and high stability. Moreover, DNAzymes, which are artificial single-stranded DNA molecules with catalytic activity, have been used for the development of many fluorescent sensors. However, DNAzymes have limited sensitivity and are vulnerable to degradation by several components, such as nucleases present in biological samples, limiting their practical applications and increasing the cost of the synthesis of more effective molecules. To meet this challenge, new cost-effective DNAzymes have been synthesized in the last few years with lower cost and increased stability to be used in fluorescent sensors for liquid biopsy applications. Since the discovery of prokaryotic Argonaute proteins (pAgos), a system of pAgos guided by short DNA has been a more versatile gene editing and sensing system with high specificity in comparison to traditional restriction endonucleases and CRISPR-Cas systems and has been used for sensor development. Magnetic isolation was used in some approaches to eliminate the background signal [13,19,20].

### 3.1. MicroRNAs (miRNAs) and PIWI-Interacting RNAs (piRNAs)

PIWI-interacting RNAs (piRNAs) are a novel class of non-coding RNAs that bind specifically to the PIWI subfamily of Argonaute proteins. They are enclosed in exosomes and are considered as potential tumor markers for cancer diagnosis. Methods for detecting piRNAs are still very limited. In this context, a novel fluorometric sensing system based on AuNPs’ quenching effect was developed for the in situ and multiplex detection of exosomal piRNAs. Dual probes were constructed. Firstly, anchor DNA (aDNA) oligonucleotides were captured by 13 nm AuNPs. Then, reporter probes that carry a Cy5 or Cy7 fluorophore were partially hybridized to aDNAs, resulting in fluorescence quenching by the AuNPs. As the hybrid AuNPs enter exosomes, the piRNAs complementary to the aDNAs remove the reporters and hybridize to the aDNA, and fluorescence is recovered. The molecules piR-651/piR-20365, and piR-651/piR-20365 were dually detected by the proposed method with good selectivity, providing a detection limit of 480 and 110 pM, respectively. Also, for exosomes, the limit of detection was 3.75 × 10^7^ and 2.50 × 10^7^ particles/μL for the two dual probes developed, respectively [8]. Moreover, a sodium-dependent and butanol-accelerated split DNAzyme fluorescent sensor with increased nuclease resistance was developed for the detection of miRNAs (miR-21) and PIWI-interacting RNAs (piRNAs-piR-20365) in extracellular vesicles (EVs) for breast cancer diagnosis. The target was hybridized to a hairpin that, in combination with Na^+^, activated a DNAzyme to cleave a molecular beacon that carried a fluorophore (FAM) and a quencher (BHQ1), generating a fluorescent signal. The DNAzyme was then used for another cleavage step inducing signal amplification. Moreover, the use of the butanol dehydration method by mixing a large volume of butanol with the aqueous sample solution increased the concentration of molecules, accelerating the reaction and strongly enhancing the signal. The method had a very low detection limit of 12 aM in a 30 min reaction and higher sensitivity than common DNAzymes [13]. A nuclease-resistant fluorogenic microgel biosensor using locked nucleic acids (LNAs) as bioreceptors was also developed for the detection of miR-let-7a at a low concentration of 1.3 fM. Molecular beacons (MBs) modified with LNAs, a fluorophore and a quencher at each end, were conjugated to microgels. Upon target addition, the MBs were opened, and fluorescence was recorded by fluorescence imaging techniques. The microgel provided effective bioconjugation, enhanced binding stability and nuclease resistance, and it was ready to use without complicating processes, enhancing its practicability. It was also hybridized quickly to the target and exhibited a high signal-to-background ratio [19].

### 3.2. Circulating Tumor DNA (ctDNA)

Exponential amplification reaction (EXPAR) combined with *Thermus thermophilus* Argonaute (*Tt*Ago) DNA polymerase was used for the rapid detection of ctDNA and more specifically for the detection of seven Kirsten rat sarcoma-2 virus (KRAS) point mutations in less than 16 min with higher specificity than traditional systems. *Tt*Ago cleaved the mutated ctDNA target to obtain specific single-stranded DNA (ssDNA) fragments (initiators) that were used for subsequent EXPAR. The EXPAR template contained two targeted repetitive sequences, a reverse complementary sequence for recognition by a nickase and a sequence for generating the initiator X. This design allowed the rapid amplification of the mutated ctDNA. The wild-type ctDNA was not cleaved by *Tt*Ago, but it was blocked for EXPAR by a mismatch between the end base of the initiator and the template. The initiator was then extended by the polymerase and cleaved upon hybridization to the template, with the producing trigger initiating another cycle of EXPAR resulting in amplification. SYBR Green fluorescent dye was used for real-time monitoring of the amplification. The system allowed detection of as low as 0.1% ctDNA, while it could also be coupled to terahertz-spectroscopy-based and lateral-flow-based detection systems with an analysis time of 16 min, with high single-nucleotide specificity [20]. Another label-free fluorescent biosensor exploited the affinity of single-stranded DNA (ssDNA) compared to double-stranded DNA (dsDNA) to Ti_3_C_2_T_x_ and the quenching effect on fluorescent CsPbBr_3_ nanosheets. An ssDNA probe was absorbed onto Ti_3_C_2_T_x_. Upon addition of the complementary target, the target was hybridized to the ssDNA probe, resulting in the formation of dsDNA that was unbound from Ti_3_C_2_T_x_, and therefore, Ti_3_C_2_T_x_ quenched the emitted fluorescence from the CsPbBr_3_ nanosheets. The method was applied for the detection of EGFR 19 Dels for non-small-cell lung cancer in ctDNA after signal enhancement through SDR and magnetic bead isolation. The method had an LOD of 180 fM [21]. Moreover, Blocked PCR and SuperSelective primer PCR were used for the detection of KRAS gene mutations in ctDNA through hybridization on DNA arrays by tailored oligonucleotides. A fluorescence (Cy5) or color (digoxigenin) label was used during PCR, with the fluorescence label giving faster analysis as it avoids the incubation step with the substrate in producing a color output [22].

### 3.3. Circulating Tumor Cells (CTCs)

In order to increase the CTC detectability in blood, a PEG-modified three-dimensional network, a AuNP-based nanovehicle and an aptamer that specifically binds to the nucleolin protein on the surface of CTCs were developed. A fluorescently labeled aptamer was complementary and hybridized to the aptamer attached to the 3D network, resulting in fluorescence quenching. In the presence of CTCs, the aptamer was bound to the CTCs, releasing the fluorescent aptamer, and fluorescence was recovered. The LOD of the aptasensor was as low as 2 cells/mL. Also, the aptasensor showed excellent specificity and sensitivity and negligible fluorescent background, allowing quantitative analysis [23].

### 3.4. Proteins

The proteins HER2, ER, PR and Ki-67, related to breast cancer, were detected in serum with detection limits of 0.37, 0.38, 0.39 and 0.39 pg/mL, respectively, using a novel colorimetric and fluorescent dual-mode immunosensor. A Cu-Zr metal–organic framework (MOF) was synthesized with catalytic peroxide-like activity. Specific antibodies to the proteins were covalently coupled to MOFs and Fe_3_O_4_ nanoparticles, forming a sandwich-type immunoassay. The magnetic separation eliminated the background signal, while a colorimetric signal was produced after the addition of the substrate o-phenylenediamine (OPD) which was catalyzed in the presence of MOFs and H_2_O_2_ to produce the yellow product 2, 3-diaminophenazine (DAP). Then, fluorescence ratiometric detection was also performed based on the internal filtering effect between DAP, which emits fluorescence at 556 nm, and carbon dots that fluorescence at 424 nm, which was quenched by DAP. The accuracy of the method reached 87.5% [1].

## 4. Electrochemical Sensors

Electrochemical sensors are famous for their very low limits of detection, simplicity, low cost and disposability, making them ideal tools for POCT platforms. A range of signal and target amplification strategies, often combined, enhance biosensor performance [24]. Electrochemical sensors employ various electrochemical methods to detect liquid biopsy biomarkers, such as chronoamperometry, differential pulse voltammetry (DPV), square wave voltammetry (SWV) and electrochemical impedance spectroscopy (EIS). Although there are no established standards linking specific biomarkers to electrochemical methods, DPV and SWV are more frequently used for miRNA detection, while EIS is commonly applied to protein and antibody biomarkers. Even though these methods differ in measurement conditions, the final readout of the electrochemical sensors is typically the current response at the working electrode surface. This current response may either decrease or increase, leading to signal-off or signal-on platforms, respectively, and is associated with the concentration of the target molecule. While signal amplification strategies typically lengthen assay times, recent studies demonstrate that they can significantly enhance sensitivity and analytical accuracy, with new methods also reducing assay duration. Key examples of each category are provided below, highlighting combinations of strategies such as surface modification, enzyme-based assays or amplification-based approaches.

### 4.1. MicroRNAs

A triple signal amplification approach using magnetic beads for target immobilization and interference purification achieved a detection limit of 6.7 aM. The miR-181b target was amplified and labeled with FAM via ligase chain reaction following the biotinylated products’ immobilization on streptavidin-coated magnetic beads. Double-stranded DNA targets were detected using an anti-FAM-HRP conjugate, generating a catalytic current on the magnetic glassy carbon working electrode. This highly sensitive biosensor was successfully applied to serum samples, making it suitable for on-field or clinical applications [25]. Leveraging nanotechnology, another work employed gold nanospheres to achieve ultrasensitive miR-9 detection. By electrodepositing a gold solution and seeding gold nanospheres on the electrode surface, a robust platform was realized, enabling probe immobilization via sulfur–gold interactions. miRNA detection occurred via EIS, eliminating the need for signal amplification or labeling. This nanostructure-based method achieved an LOD of 0.012 aM, highlighting the effectiveness of nanomaterial engineering in biosensors [26]. A sensitive electrochemical platform for detecting miRNA-31 in saliva was developed using the CRISPR/Cas12a system. Hairpin-modified magnetic nanoparticles (MNPs) were employed to amplify the miRNA target, resulting in double-stranded DNA formation on the MNPs. In the presence of the target, Cas12a was bound to the MNPs, creating a “3D nano-harvester” with high cleavage activity. This nano-harvester then cleaved and released methylene-blue-labeled hairpin probes immobilized on the electrode surface, leading to a decrease in the electrochemical signal. This method achieved detection sensitivity at the femtomolar level [27]. In another study, paper’s unique properties were leveraged to develop a paper-based electrochemical platform for miRNA-652 detection. The device featured a screen-printed electrode modified with AuNPs on office paper, onto which an anti-miRNA probe was immobilized via sulfur–gold interactions to enable selective recognition of the target miRNA. This probe was labeled with the redox mediator methylene blue (MB), and upon target recognition, electron transfer was reduced, creating a signal-off platform. Additionally, wax-patterned filter paper disks were used to pre-concentrate the sample, enhancing sensitivity by 10-fold and achieving a detection limit as low as 0.4 nM [28].

### 4.2. Circulating Tumor DNA (ctDNA)

A recent dual enzyme-assisted amplification mechanism combined with HCR was developed for the detection of ctDNA. The target ctDNA initiated HCR, generating partially double-stranded DNA sequences. These sequences served as templates for the Klenow enzyme, creating fully double-stranded DNA that acted as a recognition site for the nicking enzyme Nb.BbvCI, which then cleaved the extended sequences. The resulting fragments unfolded the hairpin probe immobilized on the gold electrode, triggering further HCR amplification. Finally, the HCR products were detected by the addition of the redox mediator MB, achieving a detection limit of 2.3 fM [29].

### 4.3. Exosomes

The properties of PbS colloidal quantum dots (QDs) were exploited for the electrochemical detection of exosomes. The QDs were used to modify the working electrode, which operates through a charge–discharge mechanism enhancing the assay’s sensitivity. CD63 antibodies were covalently bound to the modified electrode to capture exosomal membrane proteins, with the redox mediator, potassium ferricyanide, facilitating the final detection. This technique achieved an LOD of 19 particles/mL, demonstrating its capability for detecting exosomes in complex samples [30]. In another approach, a dual amplification strategy was employed for detecting exosomal miR-1246. In this work, magnetic nanoparticles (MNPs) were modified with two probes: one complementary to miR-1246 and another to CRISPR RNA. Upon miR-1246 binding to the probe–MNP conjugates, it formed a double-stranded hybrid, which was then cleaved by the DSN enzyme, releasing probe fragments. These fragments activated the CRISPR/Cas12a system, which subsequently digested immobilized MB-labeled single-stranded DNA, resulting in a decreased electrochemical signal. This dual amplification approach achieved a remarkable LOD of 50 aM, demonstrating enhanced sensitivity for detecting specific miRNA markers [31]. Complementing these approaches, an electrochemical DNAzyme-based walker combined with enzyme-catalyzed amplification was developed to detect exosomes by targeting specific surface proteins, such as MUC1 and HER2. In this setup, streptavidin-coated magnetic beads immobilized a glucose oxidase (GOx)-linked DNA strand. In the presence of MUC1 and HER2 proteins, these proteins were bound to their respective aptamers, triggering ligation reactions that bring the aptamers into proximity. This configuration facilitated hybridization with the substrate DNA strand, leading to its cleavage and the release of GOx fragments. The DNAzyme-based walker moved then along the exosome surface, enabling detection as glucose was added to the magnetic beads’ supernatant. The resulting enzymatic products were measured by an electrochemical sensor, reflecting the concentration of exosomes. This platform was also able to differentiate target exosomes from others down to 3.63 × 10^4^ particles/mL [32].

### 4.4. Proteins

A paper-based electrochemical biosensor was developed to detect the ανβ6 integrin receptor present in cancer-derived small extracellular vesicles (S-EVs). The device used a screen-printed electrode modified with AuNPs. An integrin-specific probe was immobilized onto the AuNP-modified electrode via thiol groups. Following optimization through a multidisciplinary approach, ανβ6 integrin-containing S-EVs were directly detected via EIS, achieving a detection limit as low as 0.7 × 10^3^ S-EVs/mL [33]. A sensitive electrochemical immunosensor, with a typical assay principle, was developed for detecting HER2 protein, combining the unique properties of nanodiamonds and AuNPs. The nanodiamonds were oxidized using nitric acid and deposited onto the working electrode by drop-casting. This was followed by further modification through AuNP electrodeposition. HER2-specific antibodies were then immobilized on the modified electrode’s surface. Upon adding the sample to the immunosensor, detection was carried out using EIS, achieving a low LOD of 0.29 pg/mL, with successful application to serum samples [34].

### 4.5. Circulating Tumor Cells (CTCs)

A study developed an electrochemical sensor utilizing a self-assembled DNA nanomachine, which had two main components: the extraction and enrichment of CTCs and the detection of mucin 1 on those CTCs. Initially, the focus was on optimizing the separation of CTCs from the sample. The sensing strategy involved four single-stranded DNA (ssDNA) sequences with palindromic regions that were hybridized to form a Y-shaped DNA nanosphere. An aptamer specific for mucin 1 was hybridized with one of the Y-structure sequences, disrupting the DNA nanosphere configuration. When mucin 1 was present, the aptamer was bound selectively to it, maintaining the integrity of the DNA nanosphere. Methylene blue (MB) acted as a signal reporter by embedding itself between the base pairs within the DNA nanosphere, forming a DNA-MB complex. This embedding reduced the amount of free MB, leading to a lower electrochemical signal. The sensor achieved a minimum detectable concentration of 1 ag/mL and 1 cell/mL [35].

## 5. Surface-Plasmon Resonance (SPR) Sensors

The high flexibility, label-free detection associated with biomolecular-binding-induced changes in the refractive index of incident light on the sensor’s surface due to mass changes and real-time monitoring capability of optical SPR sensors, as well as their integration with smartphone devices, make SPR sensors good candidates for useful POCT devices [36]. SPR sensors have great adaptability for liquid biopsy-based sensors and are considered as one of the gold standard techniques for the detection of biomarkers with high reliability and multiplexing potential, while they provide easy and inexpensive fabrication. The high flexibility of these sensors has driven them to POCT. New SPR sensors have been developed recently for the detection of proteins served as biomarkers in serum samples. However, label-free detection using SPR sensors lacks good detectability. Therefore, plasmonic nanomaterials, such as gold nanoparticles, are usually employed to enhance the SPR signal. For recent liquid biopsy applications, optical fibers were used as solid support for SPR sensors, and novel nanoparticles, such as polystyrene beads with a Ag/Au layer, were used for signal enhancement. Smartphone-based sensors that miniaturized the apparatus and lowered the cost have also been investigated for liquid biopsy applications. The use of proper plasmonic material was crucial for gaining good compatibility with the smartphone camera [37,38,39].

### 5.1. Proteins

An anti-crossing modulation SPR sensor was crafted for the detection of human CSPG4 protein. Specifically, a thin Au film was coated on an optical fiber tip to detect changes in the refractive index near the sensor’s surface, which are indicative of biomolecular interactions. The sensor chip was part of a system that employed a multichannel SPR spectrometer to analyze liquid samples by directing light at two sensing spots on the chip simultaneously. The anti-crossing modulation method exploited the opposite responses of reflectivity at the short wavelength dip (λS) and the longer wavelength dip (λL) that resulted in changes in the refractive index. By determining the minimum intensity at these two wavelengths and the average intensity values around these minima, the SPR output signal was defined, allowing for effective detection of protein binding events [36]. In another work, Hou reported a phage-based SPR (P-SPR) strategy for the rapid detection of carcinoembryonic antigen (CEA) in undiluted serums. The strategy included the genetic engineering of the anti-CEA single-chain variable region fragment (scFv) to be displayed on the pIII protein of the M13 phage and its modification with 5 nm AuNPs, resulting in a P-SPR probe, M13-scFv-GNPs. This probe enabled the sensitive detection of CEA by enhancing the SPR signal through the presence of GNPs. The method showed an LOD of 0.83 fM in 10 min [37].

### 5.2. Other

**Figure 3:**
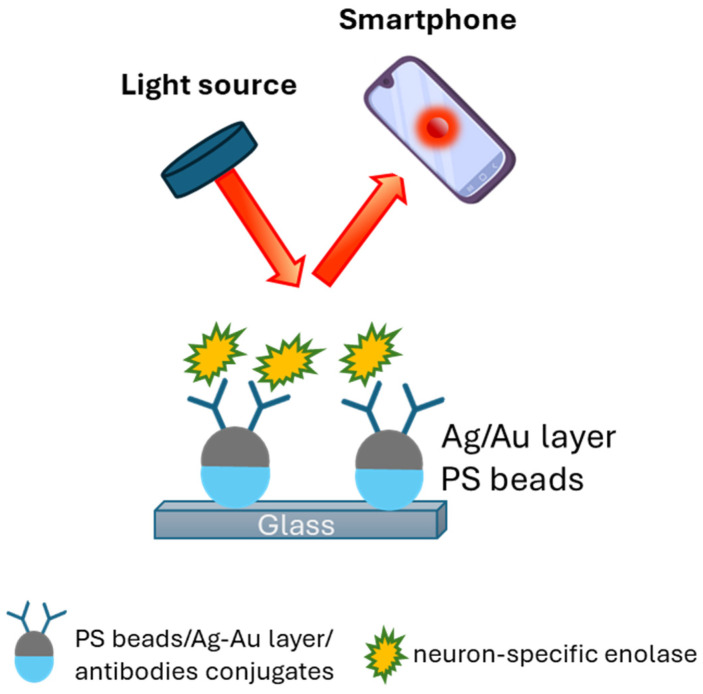
An SPR sensor based on polystyrene beads with a Ag/Au layer for signal enhancement coupled to antibodies for specific detection of neuron-specific enolase [38].

An SPR sensor was developed for the detection of the tumor marker neuron-specific enolase (NSE). Polystyrene beads with a Ag/Au layer (silver nanodome arrays) for signal enhancement were coupled to antibodies specific for NSE and were immobilized onto a glass surface. The signal was recorded by a smartphone with subsequent image processing (Figure 3). The sensor had an LOD of 270 pM [38].

In an alternative route, an SPR-based sensor for the detection of cancer cells in multiple kinds of samples was established. This sensor utilized a bi-metallic layer coated on the prism surface, followed by a nitride layer (composed of materials like AlN, GaN, InN and Si_3_N_4_), differentiating for each type of cancer cell. When p-polarized light was directed through the prism and touched the prism–metal interface, it underwent total internal reflection. At a specific angle of incidence, known as the resonance angle (θr), the light induced collective oscillations of the carriers in the metal, resulting in a minimum in the reflected light intensity. This phenomenon allowed for the detection of changes in the refractive indices of the cells placed on the nitride layer, which differ between normal and cancerous cells [39].

## 6. Surface-Enhanced Raman Scattering (SERS) Sensors

SERS is an optical technique that has been widely used for the ultrasensitive detection of biomarkers in liquid biopsy due to bright signals, large signal enhancement, narrow spectra widths and multiplexing capability. Raman scattering involves the inelastic scattering of photons by matter and is detected mainly by a photomultiplier. Label-free detection is one of the main advantages of SERS. The use of mesoporous metal nanostructures, such as gold films, induces higher surface area for enhanced loading capacity of recognition biomolecules, and it strongly enhances the signal through surface-enhanced light scattering [40]. SERS substrates with strong absorption capability were introduced for complex and viscous body fluids with high heterogeneity, such as saliva. Multivariate statistical methods and machine/deep learning technologies were exploited for the interpretation of large data and spectral features of Raman spectra, enhancing clinical sensitivity, specificity and accuracy [9,10]. Currently, SERS sensors have been developed for CTC and cancer metabolite detection. Raman scattering also provided a fingerprinting of the metabolic profile of body fluids based on molecular vibrational and rotational information. Finally, liquid-state SERS detection in body fluids compared to steady-state detection was also examined.

### 6.1. Circulating Tumor Cells (CTCs)

Ahmed et al., in 2024, developed nanostructured mesoporous gold films coated with biotinylated BSA, streptavidin and a capture antibody (anti-EGFR) to selectively capture lung CTCs on peripheral blood mononuclear cells. SERS nanotags, which were AuNPs coated with specific thiol monolayers, were conjugated to detection antibodies and contained Raman reporters, like 2,3,5,6-tetrafluoro-4-mercaptobenzoic acid (TFMBA). When excited, SERS nanotags emitted distinct signals that corresponded to the expression of specific immune checkpoint proteins (PD-L1, CD276, B7H4, and CD80), all related to lung cancer. The analytical results showed that the platform could capture and analyze individual cancer cells successfully, versus bulk analysis methods, and could have high potential in the field of personalized therapeutics. The ultimate aim was to identify and monitor the immune checkpoint proteins (ICPs) that play a vital role in the immune response against cancer [40]. In another work, a SERS-functionalized L-MISC (lung metastasis-initiating stem cell) nanosensor was developed that captured blood cells on the sensor’s surface, and Raman scattering profiles were recorded. Cancer stem cells are responsible for metastasis and address the heterogeneity in many types of cancer and were chosen here as promising biomarkers to detect metastasis. By further employing a machine learning model, this sensor emerged as a promising, rapid and low-invasive diagnostic tool for detecting lung cancer metastasis in 5 μL blood samples with 100% clinical sensitivity [41].

### 6.2. Cancer Metabolites

Linh et al., in 2024, in their recent work, focused on the SERS enhancement capabilities of 3D plasmonic hexaplex paper sensors (3D-PHPs), which played a crucial role in their sensitivity and effectiveness for cancer screening. The enhancement mechanism relied on the localized surface plasmon resonance (LSPR) effect, which involved nanostructures of gold with sharp tips (spikes) strongly amplifying the Raman signals of molecules near the sensing surface. The sensor’s SERS activity was experimentally validated using methylene blue as a model Raman reporter molecule. Under 633 nm and 785 nm laser excitations, the 3D-PHPs activated high SERS intensities, with a limit of detection of 0.13 nM and 1.63 nM, respectively. The sensors were tested directly on saliva samples, where each sample was simply dropped onto the substrate, with no additional treatment needed, in order to analyze lung cancer metabolites through SERS fingerprinting. A logistic-regression-based machine learning model was also developed for the automated classification of patients with 91.2% sensitivity, 80.2% specificity and 87.5% accuracy [10]. Another study focused on the development of a non-invasive, highly sensitive and highly specific cancer diagnosis method based on SERS, using whole human urine samples. This method combined a 3D evolutionary gold nanoarchitecture (3D-EGN) platform with machine learning techniques to classify cancer types. The platform was fabricated using a combination of a nanoporous gold structure (AuS), gold nanoparticles, and gold nanolamination to form a multilayered sponge-like structure. The 3D-EGN exhibited strong electromagnetic fields and numerous hotspot regions, essential for detecting trace analytes in urine in a liquid state, using SERS. After the identification of these metabolites, machine learning (logistic regression and Convolutional Neural Network) was applied to classify urine samples as normal or cancerous. The model achieved an accuracy of 95.6%. When using malachite green as a Raman reporter, the platform showed a limit of detection of 1.23 nM [9].

## 7. Clustered Regularly Interspaced Short Palindromic Repeats (CRISPR)-Based Sensors

CRISPR/Cas-associated protein is a novel technique that provides enhanced selectivity and specificity compared to other methods and is considered as the next generation of molecular diagnostic technologies. It provides exceptional recognition specificity and efficient signal amplification. CRISPR/Cas cleaves selectively non-specific ssDNA reporters upon circular RNA (crRNA) target recognition. The endonuclease activity of Cas proteins is activated exclusively when the target DNA is perfectly hybridized with the crRNA. The target is recycled, and numerous signal reporters are produced, leading to strong signal enhancement [42]. This technique was highly adopted for ctDNA detection and less for exosomes and proteins. Recently, a CRISPR/Cas12a system was integrated with nanomaterials for enhanced binding capacity and fluorescence readouts using molecular beacons that contain a fluorophore and a quencher, fluorescent reporter molecules, fluorescent dyes or chemiluminescent reporters. This system was also combined with amplification techniques such as CHA, DSN and HCR for further a signal increase, avoiding traditional nucleic acid amplification techniques. DNA tetrahedron-modified magnetic beads have also been used for exosome capturing and detection due to their excellent stability in body fluids and high capture efficiency, followed by magnetic separation to enable exosome enrichment and detection of low concentrations of exosomes. Aptamers were also utilized here to capture the surface proteins on exosomes. The utilization of AuNPs and dark-field microscopy was also exploited, achieving low detection limits.

### 7.1. ctDNA and Cell-Free DNA (cfDNA)

An et al., in 2023, detected ctDNA in serum samples using CRISPR/Cas12a and metal–organic framework (MOF)-based fluorescent labels. MOFs are novel nanomaterials with high surface and porosity, which lead to increased loading of molecules and thus signal enhancement. Specifically, amino-functionalized MOFs were first synthesized and loaded with Cy5 dye and then blocked with hairpin DNA to retain the fluorescent molecules. These labels were grafted in APTES- and streptavidin-modified magnetic beads, using an ssDNA linker, to create MB-ssDNA-MOF probes. So, when the target ctDNA was present, it activated the CRISPR/Cas12a system, leading to the cleavage of the ssDNA linker. The released fluorescent labels were collected via magnetic separation, and the fluorescent intensity was measured to determine the ctDNA concentration. Due to the fluorescent signal enhancement from the MOF-based labels in combination with CRISPR/Cas12a, the method achieved an LOD of 5.6 fM, increasing the detectability four times in magnitude compared to the existing methods. Finally, the method provided a successful distinction between lung cancer patients and healthy individuals [43].

**Figure 4:**
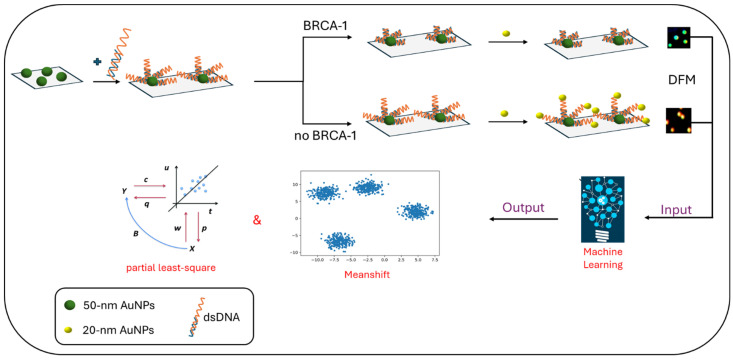
A CRISPR/Cas12a system based on AuNPs and dark-field microscopy (DFM) for detecting BRCA-1 mutations related to breast cancer. Meanshift and partial least-square algorithms were used for signal interpretation [45].

A CRISPR/Cas12a-based biosensor was developed in order to analyze cfDNA for BRCA-1 mutations without amplification. When BRCA-1 was present, the Cas12a system activated the DSN, which facilitated signal recycling in the H1@MBs probe. As a result of the signal recycling, there was a difference in the fluorescence signal of the H1 probe. The presence of cfDNA also affected the absorbance of the H2@AuNPs probe by triggering a reaction that caused AuNPs to aggregate, which was visually confirmed by a color change in the solution. The combined fluorescent and colorimetric signals provided a dual-mode detection mechanism, allowing for the effective identification of cfDNA without the need for amplification steps typically required in other methods. The authors reported improved accuracy and selectivity, as well as an LOD of 1 pM (colorimetric) and 365 aM (fluorescence) [44]. In a different approach, Li and his team, in 2024, created a sensor that utilized AuNPs and dark-field microscopy (DFM) to detect BRCA-1 in cell lysates (Figure 4). When cfDNA was present, it was bound to CRISPR/Cas12a complexes, activating the CRISPR/Cas12a system. This activation led to the cleavage of the dsDNA that was conjugated to 50 nm AuNPs1. Then, with the addition of 20 nm AuNPs2, there were no free –SH groups to connect with, so no aggregation occurred, and green scattering could be observed under DFM. In contrast, if no BRCA-1 target DNA was present, the formation of AuNPs1-dsDNA-AuNPs2 aggregates happened, which showed red or yellow scattering points. The scattering signals were processed with Meanshift and partial least-square algorithms to quantify the cfDNA concentration based on the ratio of monomer to aggregated AuNPs. The method achieved high sensitivity with a detection limit of 0.081 fM for BRCA-1 in 40 min [45].

### 7.2. Exosomes

An integrated magneto-fluorescent (iMEX) nanosensor was developed for the detection of PD-L1-positive exosomes derived from NSCLC cell lines. The nanosensor employed magnetic beads functionalized with DNA tetrahedral lipid probes (MBs@DTLP) for rapid and enhanced capturing of exosomes from plasma samples. A bifunctional aptamer (PD-L1-T) was specifically bound to the exosomal membrane proteins, forming sandwich complexes that triggered a CHA reaction. The CHA reaction generated multiple H1/H2 duplexes, which activated the CRISPR-Cas12a system for the trans-cleavage of a fluorescent reporter substrate (FAM-TTATT-BHQ1), resulting in amplified fluorescent signals. The nanosensor achieved a dynamic range of 2.86 × 10^3^ to 2.86 × 10⁷ particles/μL, with a detection limit of 1.71 × 10^3^ particles/μL within 1.5 h [46]. In another work, a CRISPR/Cas12a and aptamer-chemiluminescence-based analysis (CACBA) method emerged for the detection of EpCAM- and MUC1-positive exosomes in plasma specimens collected from patients diagnosed with breast cancer. The CACBA method utilized CRISPR/Cas12a technology combined with aptamer-based detection. When the target exosome was bound to its corresponding aptamer, it activated the Cas12a enzyme, which then cleaved an ssDNA molecule modified with a fluorophore and a quencher. Upon activation, the fluorophore was released from the quencher, resulting in fluorescence recovery producing a measurable signal. This signal was proportional to the concentration of the targeted exosomes, allowing for specific and sensitive detection ranging from 2.86 × 10^3^ to 2.86 × 10^7^ particles/μL, with a detection limit of 1.71 × 10^3^ particles/μL. The method exhibited higher accuracy and robustness than existing methods [47].

### 7.3. Proteins

A dual-aptamer HCR method that exploited CRISPR for high selectivity was used for detecting the proteins EpCAM and HER2, which are associated with tumor extracellular vesicles (TEVs). The method employed a dual-aptamer-based AND logic gate for the specific recognition of TEVs, where aptamers bound to target proteins on TEVs formed a complex only if both targets were present. The AND logic triggered an HCR that amplified the signal by creating long-nicked ssDNA, which was then recognized by the CRISPR-Cas12a system. The Cas12a enzyme selectively cleaved a fluorescent reporter probe, releasing a fluorescent signal that was proportional to the concentration of TEVs. In clinical plasma samples, the method showed 100% accuracy, a very low LOD of 3.3 × 10^2^ particles/μL, rapid analysis, simplicity and high specificity. By changing the aptamers, the sensor could be applied for the detection of other TEVs or achieve multiplex analysis [42].

## 8. Rolling Circle Amplification (RCA)-Based Sensors

RCA is a traditional signal amplification technique that is used for the isothermal amplification of specific nucleic acid sequences with high specificity. It is performed by employing a single-strand primer probe and DNA polymerase to create a lengthy nucleic acid strand along a circle template. The long-stranded products contain repeat sequences complementary to the cyclic template; hence, the product sequence may be modified by changing the template sequence. Aptamers were also chosen here for exosomal protein detection. Aptamers were used to convert the protein signal on the surface of the exosomes to a DNA signal that can be further amplified by RCA, increasing the final signal output. Fluorescent readouts were combined here with RCA.

### 8.1. Circular RNAs (circRNA)

A reverse transcription reaction in combination with a hyperbranched rolling circle amplification reaction (RT-HRCA) was exploited for the detection and quantification of circulating RNA of the circHIPK3 gene with high selectivity against its linear RNA sequence. The detection/quantification of amplified products was performed using a digital droplet microfluidic device, the LAMP fluorescent intercalating dye and a fluorescence microscope, providing an LOD of 6.6 aM [48].

### 8.2. Exosomes

**Figure 5:**
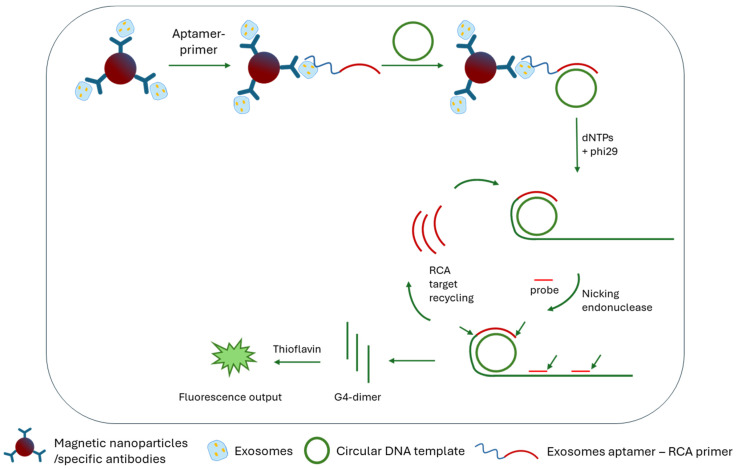
Detection of exosomes through RCA using an aptamer specific for exosomes and dimer-G4/thioflavin-based fluorescence output [49].

A label-free immunoassay was developed based on guanine quadruplex (dimer-G4) signal units produced by cutting-mediated exponential rolling circle amplification (CM-ERCA). Exosomes were first enriched via immunomagnetic separation. Then, molecular recognition took place via an antibody–exosome–aptamer immunocomplex through the specific binding of the aptamer-primers to the exosomes. CM-ERCA was then triggered by the hybridization of the aptamer primer on the exosomal surface to a circular DNA sequence. During RCA, numerous dimer-G4 units were produced, which led to increased fluorescence upon the addition of thioflavin T (ThT) (Figure 5). The dimer-G4/ThT system strongly enhanced the detectability due to its high quantum yield. Thus, the low detection limit achieved, 2.4 × 10^2^ particles/mL, was due to the fluorescence enhancement of dimer-G4 signal units [49]. Lung-cancer-related exosomes were also detected by targeting the protein cell death ligand 1 (PD-L1) that was located on the surface of the exosomes. The protein was recognized by a specific aptamer that initiated HCR. The Cu^2+^ intercalated the dsDNA HCR product, and upon calcein addition, fluorescence was emitted, achieving signal amplification and allowing quantification. The method provided a low LOD of 100 particles/mL [50].

### 8.3. Proteins

Guo et al., in 2024, utilized RCA and a functionalized MOF integrated with dual-aptamer recognition for the sensitive detection of cancer low-abundance delivered extracellular vesicles in serum samples. The method began with the functionalization of the MOF with aptamers that specifically recognized surface proteins on EVs (such as CD63 and PTK7), which allowed the selective capture of EVs from complex samples. Then, a second aptamer was introduced which targeted another protein on the EV surface. This triggered the RCA process, where a circular nucleic acid template and DNA polymerase were used to produce long nucleic acid strands that contained repetitive sequences. Finally, the amplified products formed G-quadruplex structures that were bound to the fluorescent dye thioflavin T (ThT). The fluorescence intensity was correlated to the concentration of the EVs, allowing for quantitative analysis. The proposed biosensor showed an LOD of 2.2 × 10^4^ particles/μL and an ability to successfully distinguish EV expression in the plasma of patients from that of normal subjects. MOFs are 3D nanomaterials produced by the self-assembly of metal ions with organic ligands that have a large surface area, variable size, available functional groups and chemical stability, being suitable candidates for increased capturing of EVs [51].

## 9. Lateral Flow Assays (LFAs)

Paper-based devices are one of the key devices for point-of-care testing (POCT) ‘on-site’ due to their simplicity, cost-effectiveness, portability, versatility and disposability [52,53]. Lateral flow assays are categorized as lateral flow immunoassays (LFIAs) in a sandwich or competitive format that detect proteins or other antigens using specific antibodies and nucleic-acid-based ones that detect specific nucleic acid sequences, usually after amplification. Aptamers have recently replaced antibodies in LFIAs to lower the cost of the analysis and increase specificity [54]. LFAs have also benefited from the use of nanomaterials with excellent optical properties, increasing their specificity and detectability. LFAs are strip-type biosensors that consist of four distinct parts, namely the immersion pad, the conjugate pad, the diagnostic membrane and the absorbent pad. The sensing areas of the LFAs are constructed as zones by spraying proper recognition molecules onto the diagnostic membrane. Nanoparticles are conjugated to other recognition molecules serving as reporters to produce the optical signal. Colored, fluorescent or luminescent and SERS-based nanoparticles are used to produce the optical signal that is visualized by the naked eye or special strip readers. The sample travels along the strip through capillary forces, while the reporters accumulate in the sensing areas of the strip, forming visual lines. The colored nanoparticles are visualized as color lines, or a change in color is observed after aggregation on the sensing areas. Fluorescence is measured directly or through energy transfer to other fluorescent nanoparticles or quenching molecules. The combination of novel and unique nanoparticles and nanomaterials has strongly influenced the specificity and detectability of LFAs. The addition of Raman tags to common colored nanoparticles, such as gold nanoparticles, has increased the detectability of bare nanoparticles. Recently, LFAs have been mainly developed for miRNA detection. Novel nanoparticles were utilized for signal generation. For miRNA detection, CHA and stem-loop RT-PCR were chosen as amplification strategies. Finally, aptamers were immobilized on the test zone of LFAs to capture and detect exosomes by aptamer-functionalized fluorescent microspheres and MnO_2_ nanosheets exploiting FRET.

### 9.1. MicroRNAs

A multimode LFA was established for fluorescent, colorimetric and SERS-based detection of microRNA-21 in saliva and serum samples, using core–shell, DNA functionalized, Au-DTNB@Ag nanoparticles as reporters. In fluorescent analysis, the analyte was bound to the Au-DTNB@Ag nanoparticles, and the accumulation of the nanoparticles on the test line led to quenching of the fluorescence from Upconversion Nanoparticles (UCNPs) through the Förster resonance energy transfer (FRET) process. Similarly, when the target–Au-DTNB@Ag complex was bound to the ssDNA probe immobilized on the test line, the nanoparticles aggregated, leading to a local increase in their concentration, which altered the optical properties and resulted in a quantitatively visible color change. Finally, the SERS signal of DNTP (5,5-dithio-bis-(2-nitrobenzoic acid)) molecules increased in correlation with the miR-21 concentration when the same aggregation occurred. UCNPs are novel fluorescent nanoparticles with a narrow emission band, high stability and long lifetime. Multimode signals on the LFA, such as FRET and SERS, increased the accuracy of the detection. This system overcame the need for complicated amplification procedures and offered a dynamic range in the range of 2 nM to 1 fM [53]. In a totally different approach, Xu et al., in 2023, developed a ratiometric strip sensor that utilized a tetrahedral DNA capture probe and CHA reaction for the detection of exosomal miR-150-5p in HK-2 cells. The tetrahedral probe was basically an immobilized barcoded tetrahedron structure that served as a scaffold for capturing probes. When the target was present, it triggered a CHA that led to the formation of duplexes, which were then bound to streptavidin-modified gold nanoparticles (SA-AuNPs) via biotin–streptavidin interaction. This binding resulted in the accumulation of AuNPs, producing a visible red line in the test zone of the strip. The excess SA-AuNPs moved upwards, where they were captured by a biotinylated tetrahedron at the control line. As the concentration of the target microRNA-150-5p increased, the test line signal intensified, while the control line signal decreased, allowing for a quantitative assessment based on their relative intensities. This method showed an LOD of 58.9 fM [54]. Moving forward, a portable fluorescent LFA for detecting the myocardial biomarker microRNA-133 in serum for POCT was reported. The analytical process was as follows: The target was firstly mixed with two hairpin DNA probes, H1 and H2, where H1 was hybridized to the target, opening its stem-loop structure and triggering the CHA reaction to form H1/H2 duplexes. These duplexes activated the Cas/crRNA system, which was pre-mixed with biotin–DNA–digoxin probes, allowing for trans-cleavage activity. Then, the reaction mixture was added to the running buffer of the LFA strip. The membrane of the LFA had immobilized streptavidin on the test line and IgG antibody on the control line. The biotin–DNA–digoxin probes were bound to streptavidin and SiO_2_@DQD nanoparticles, providing a strong fluorescent signal. The excess of the nanoparticles was captured by the immobilized IgG, resulting in a visible signal at the control line, indicating that the test performed correctly. After 15 min, fluorescence intensity was measured using a fluorescent strip reader. This method allowed for direct quantification of microRNAs with high specificity and increased detectability, giving an LOD of 0.32 fM and recovery rates of 99.65–102.38% [55]. Finally, an LFA was combined with stem-loop RT-PCR and AuNPs, with the purpose of detecting miRNAs in urine. As a model, miR-21 and miR-let-7a were used; they were spiked in urine samples collected from healthy individuals, isolated with either a commercial kit or polystyrene beads and amplified with stem-loop RT-PCR. The amplified biotin-labeled miRNAs were then hybridized to specific DNA probes that carried a poly-dA tail and applied on the strip. The hybrids were captured at the test zone of the strip by immobilized poly-dT sequences through dA/dT hybridization. AuNPs conjugated to an anti-biotin antibody were accumulated at the test zone through anti-biotin antibody–biotin interaction, forming a red spot and indicating a positive result. The formation of a second red spot in the control zone of the strip by immobilized biotinylated albumin protein confirmed the proper strip’s functionality. This method was rapid, detecting as few as 10^2^–10^3^ copies of miR-21 and 10^2^–10^4^ copies of miR-let-7a spiked in urine samples. The developed strip was also universal and could be applied for the detection of any other miRNA [56].

### 9.2. Exosomes

A novel LFA was developed for the detection of exosomes in serum. The detection was based on aptamer-functionalized fluorescent microspheres (FMs) and MnO_2_ nanosheets. The MCF7 breast cancer cells were used as a model. Aptamer-functionalized FMs were deposited on the test line of the strip, and upon the addition of MnO_2_ nanosheets, the fluorescence of FMs was quenched by the nanosheets due to FRET. In the presence of exosomes, the exosomes were bound on the test line to the immobilized aptamers, hindering FRET by the MnO_2_ nanosheets. Concentrations as low as 2.5 × 10^3^ particles/mL could be detected by the proposed LFA. The dual-signal platform reduced the false positive results, providing rapid and direct detection of exosomes, avoiding tedious pre-treatment of the samples and proving the high potential for POCT [52].

## 10. Other Sensors

A label-free microfluidic biosensor was developed for the detection of metastatic risk through urine biopsy. A smartphone-based sensing system visualized the nematode movement of urine samples using optical microscopy. Readouts were available within 60 min of sample infusion, and low sample volume (1 mL) was required [57].

## 11. Artificial-Intelligence-Integrated Sensors

The integration of biosensors with artificial intelligence (AI) tools, such as machine learning (ML) and deep learning (DL), or other smart techniques, has enabled advances in intelligent point-of-care diagnostics and increased sensing performance. The use of AI tools has resulted in a new generation of biosensors and set the basis for a new era of precision and personalized medicine. These tools provided big data analysis and pattern recognition, resulting in automative categorization of the biomarker(s) present in a sample. Moreover, AI/ML tools enabled the prediction of the interaction of specific biomarkers with the sensing area of the biosensors, minimizing optimization experimental steps [58]. Towards the harnessing of AI and ML, Li et al., in 2024, used machine learning algorithms for the analysis of the images obtained by single-nanoparticle dark-field microscopy (DFM) [45]. In order to analyze complex Raman spectral features obtained from multiple components in body fluids, machine learning was applied for more accurate and easier analysis assisting SERS detection. Machine-learning- and/or deep-learning-assisted SERS detection was exploited for the analysis of complicated Raman spectra by Linh et al. in 2024 for machine-learning-assisted early-stage lung cancer screening depending on the profile of saliva samples [10], by Premachandran et al. in 2024 for the detection of lung cancer metastasis from blood using an L-MISC (lung metastasis-initiating stem cell) nanosensor based on the metastatic profile [41], and by Ja’farawy et al. in 2024 to obtain the metabolite profile of urine samples [9].

## 12. Discussion

Liquid biopsy has emerged as a significant diagnostic tool for early detection of cancer and prognosis. Liquid biopsy aims towards personalized medicine and point-of-care testing in order to increase the effectiveness of diagnosis and especially the effectiveness of treatment. Several biomolecules in various body fluids have already been targeted as potential liquid biopsy markers. As liquid biopsy offers the capability of frequent and continuous sampling, e.g., blood samples, and as most of the targeted biomarkers have a short lifetime in body fluids, ranging from a few minutes up to 2–3 h, the liquid biopsy approach provides real-time monitoring of tumor progression or treatment results. Compared to tissue biopsy, which is a highly invasive technique, liquid biopsy is less invasive, also providing information about the heterogeneity of a tumor, and therefore, treatment can be more effective. As for urine or saliva samples, liquid biopsy represents a non-invasive approach.

**Figure 6:**
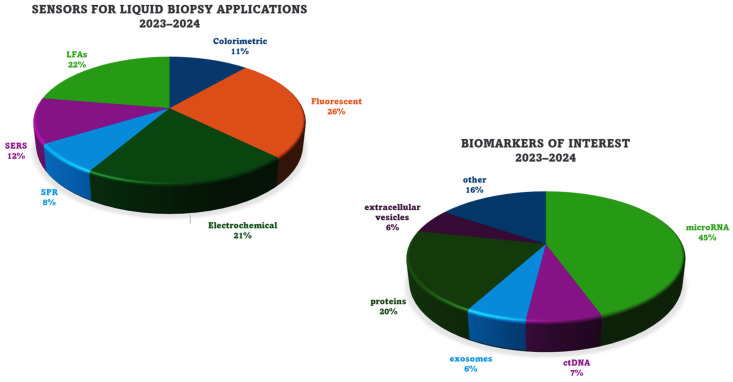
An overview of the sensors and biomarkers of interest that have emerged for cutting-edge liquid biopsy towards point-of-care testing.

In the research community, there is still an enormous effort to develop robust sensors and methods that can be finally used in routine diagnosis. There are some drawbacks, such as robustness, detectability, reproducibility, specificity and practicability, that have to be met in order for liquid biopsy to succeed in cancer diagnosis and prognosis. Emerging sensors for cutting-edge liquid biopsy applications include colorimetric, fluorescent, electrochemical, SPR and SERS sensors, as well as LFAs. As liquid biopsy biomarkers, microRNAs, ct/cfDNA, exosomes, EVs, proteins and other molecules such as metabolites have been selected. Figure 6 provides an overview of the recently developed sensors and the cancer-related biomarkers, which are presented as abundances. As most of the targeted biomarkers can be found in extremely low concentrations, amplification techniques, either for the target itself or for signal enhancement through target recycling or the implementation of nanomaterials, are used in the majority of the developed sensors.

Colorimetric sensors have the advantage of easy interpretation of the visual outcome, and no expensive or special instrumentation is needed for signal recording, enabling on-site detection and enhancing the potential for POCT. In order to produce a color signal, enzymes, nanozymes and DNAzymes are used, as well as colored nanoparticles/nanostructures. The use of novel nano-enzymes has increased the stability and versatility of the developed sensors compared to natural enzymes. Fluorescent and electrochemical sensors have increased the detectability of colorimetric sensors. By exploiting FRET phenomena, homogeneous assays eliminating the incubation and washing steps were developed, thus reducing the total analysis time. SPR and SERS sensors enable label-free detection and minimize the procedure steps and the diagnostic time. SERS exhibit ultrasensitive detection, while detectability is still an issue for SPR sensors that has to be addressed. These sensors, in order to account for POCT, must be supplied as portable devices. SPR sensors have also the great advantage of real-time monitoring. Electrochemical sensors and LFAs provide simplicity, portability, versatility and rapid analysis, being valuable tools for liquid biopsy diagnostics. Electrochemical sensors are also able to provide accurate quantitative results, but LFAs can only result in semi-quantitative estimation. The nanomaterials used for signal enhancement included mainly AuNPs and other gold nanostructures, graphene oxide and metal-based nanosheets mostly for fluorescence quenching, MOFs nanosheets again for FRET, Ag nanoparticles, a DNA nanomachine in one report and magnetic nanoparticles. G-quadruplex structures combined with colorimetric or fluorescent substrates were exploited very often in the reports for signal production (color or fluorescence). Aptamers have also been used in sensors as a cost-effective alternative to antibodies, exhibiting high affinity and specificity for the target, easy synthesis and increased stability [16,17].

Among all recently reported sensors that are presented in Table 1, the most rapid ones are the fluorescent sensors, and the most sensitive are the electrochemical sensors. Colorimetric ones had an analysis time of 20 min–2.5 h, 0.65 fM LOD for ctDNA, 7 pM LOD for miRNAs and 37–380 particles/μL for exosomes. Fluorescent sensors provided an analysis time of 12 min–1 h or 4 h, in one report, 25 aM–180 fM LOD for ctDNA, 110–480 pM for piRNAs, 2 cells/mL for CTCs and 0.3–0.4 pg/mL for proteins. Electrochemical sensors had an analysis time of 200 s in one reported study or 45 min–5 h and LODs of 0.012 aM–3.5 fM for miRNAs, 1 cell/mL for CTCs, 20 pM for antibodies, 19–24 cells/mL for exosomes and 0.3 pg/mL for proteins. As for SPR sensors, an analysis time of 5–100 min was recorded, but analysis time and LODs were not provided in most reports. An LOD of 270 pM was achieved for neuron-specific enolase, and 0.83 fM for carcinoembryonic antigen. SERS sensors exhibited a single-cell detection for metastasis-initiating stem cells and 0.13–1.63 nM for saliva metabolites. Unfortunately, analysis time was not provided for all reports. For CRISPR-based sensors, the analysis time was 20 min–4 h, and they provided an LOD of 0.086 fM–86 nM for ct/cfDNA and 1.45–3.7 × 10^2^ particles/μL for exosomes or EVs. RCA-based sensors had an analysis time of 2–3 h, 6.6 aM LOD for circular RNA and 100–2.2 × 10^4^ particles/mL for exosomes and EVs. Finally, a total analysis time of 20 min–2 h was achieved, as well as an LOD of 0.32 fM–2 nM for miRNAs and 2.5 × 10^3^ particles/mL for exosomes.

The sensors that achieved the best performance in terms of detection capability were as follows: (i) 6.6 aM of circular RNA after RCA amplification; (ii) 0.012 aM of miR-9 using differential pulsed voltammetry (DPV) in an electrochemical sensor along with gold nanostructures; (iii) 6.7 aM of miR-181b using triple signal amplification and amperometry with an electrochemical sensor; (iv) 2.18–10 aM of miR-155 and miR-21 based on square wave voltammetry (SWV) and duplex-specific nuclease (DSN); (v) 45 aM of miR-21 based on isothermal amplification and SWV detection; (vi) 50 aM of miR-1246 after amplification using CRISPR/Cas-12a, DSN, magnetic nanoparticles and SWV; (vii) single-cell detection of metastasis stem cells by SERS; (viii) 1 CTC/mL using DPV-based electrochemical sensor and a DNA nanomachine/Y structure; (ix) 2 CTCs/mL by FRET and AuNPs; (x) 2.5 particles/mL for exosomes using fluorescent microspheres conjugated to aptamers; (xi) 19 particles/mL for exosomes using a DPV-based electrochemical sensor and QDs; (xii) 100 particles/mL of exosomes after RCA amplification and fluorescence readout; (xiii) 37–50 particles/μL of exosomes by a colorimetric sensor based on HCR/G-quadruplex for color signal output or TdT-assisted AuNP aggregation leading to a color shift.

Finally, SERS has been integrated with machine learning technology for the effective interpretation of spectral features and patterns and the correct classification of a large amount of data. Nowadays, the integration of AI and image analysis tools with analytical methods, biosensors and sensing devices is the state of the art in sensing technology. The adaptation of these tools has transformed the landscape of scientific research. Sensor development has greatly benefited from AI. Machine learning is one of the most popular methods of AI that use algorithms for the analysis and interpretation of specific patterns/profiles. The aim is to enhance the accuracy of data interpretation without human intervention, avoiding personal estimations that lead to questionable results. AI tools can classify negative and positive samples more precisely, eliminating false negative/positive results and enhancing the whole performance of analytical methods and sensors, thus enhancing a diagnosis [59,60,61,62,63].

## 13. Limitations and Drawbacks

Liquid biopsy sensors face several significant limitations in their application in clinical routine. One of the primary challenges is the low abundance of biomarkers, particularly in the early stages of disease or during relapses, and the high level of interfering biomolecules, such as DNA, RNA, proteins and vesicles. This low abundance necessitates highly sensitive detection techniques, often requiring preconcentration strategies or signal amplification methods, which can complicate sensor design and extend analysis times. Additionally, biomarkers like nucleic acids and proteins are often unstable in biological samples, with short half-lives and high degradability, further complicating their detection, and the occurrence of a large number of biomarkers secreted by normal cells interferes with detection. For instance, colorimetric sensors rely on detecting visual signals, but their sensitivity may be insufficient for detecting slight color changes at low biomarker concentrations. Similarly, Raman scattering techniques like SERS, while highly sensitive, produce weak signals without enhancement, requiring the use of plasmonic nanostructures (e.g., gold or silver nanoparticles). For EV detection, the direct particle counting method is not recommended, because the identification of EVs is required.

Beyond biomarker challenges, limitations also arise from the sensors themselves, particularly concerning specificity and multiplexing capabilities. Many sensors struggle to distinguish between similar biomarkers due to their structural or sequence similarities, leading to false positive results or reduced accuracy. This lack of specificity can significantly undermine the reliability of diagnostic results, especially when distinguishing between closely related cancer types or identifying early-stage disease. Furthermore, while multiplexing sensors’ ability is a key goal for liquid biopsy sensors, many existing technologies face challenges in achieving effective multiplex detection. Sensors such as SPR and electrochemical methods may become less effective when trying to detect multiple biomarkers at once due to interference between signals or the need for complex surface modifications. Additionally, fluorescent sensors can suffer from cross-talk between different fluorescent labels, further complicating multiplex assays. Despite advances in AI and signal enhancement strategies, overcoming these issues of specificity and multiplexing remains crucial for improving the diagnostic potential of liquid biopsy sensors in clinical applications. Sensing systems may have tedious fabrication or complex experimental procedures for signal enhancement that also limit their practicability in routine analysis. The stability of the biosensors is also an issue that has to be addressed so that liquid biopsy sensors can be adopted for clinical routine diagnosis. As special and expensive instrumentation is still used in some cases, sensing systems must be directed towards simple and portable apparatuses.

## 14. Conclusions and Future Perspectives

Liquid biopsy is a promising diagnostic tool with advantages over traditional tissue biopsies in terms of simplicity, convenience, rapid analysis and cost-effectiveness. Enhanced POCT and early diagnosis are based on the sensitive, selective and real-time monitoring of a disease. If multiple biomarkers that are released from a tumor can be detected in a single sample, information about tumor heterogeneity can be available, increasing the accuracy of tumor diagnosis. The ability of clinicians to obtain frequent samples from patients allows for the monitoring of tumor progression, the effectiveness of tumor treatment and the early diagnosis of possible recurrence and metastasis. However, liquid biopsy still has many drawbacks that have to be addressed to improve detection efficiency. Detecting biomarkers at very low abundances and accurate quantification are still challenging, along with multiple analyses for the simultaneous detection of different biomarkers in a sample and, if possible, for several types of cancer to obtain more information about the tumor, making the applied treatment more effective. Moreover, for EV or exosome detection, a single protein is usually targeted. Due to the great heterogeneity of proteins on the surface of these particles, the existing methods do not provide high accuracy in diagnosis. Therefore, improving the detectability and multiplicity of existing sensors is a key for advanced liquid biopsy.

Further research is imperative for the effective integration of liquid biopsy biomarkers into point-of-care cancer testing. Existing biosensors are restricted to identifying only specific known biomarkers in a single format. However, clinical diagnoses often require the detection of multiple targets, especially in cases involving unknown mutations, in order to provide enhanced personalized medicine and effective individual treatment for each patient. This review reported the recent advances in sensors with liquid biopsy applications, focusing on the targeted biomarkers, amplification techniques for signal enhancement, analysis time and limit of detection.

## Figures and Tables

**Table 1:** Overview of recently reported sensors for various biomarkers, including their principles, associated diseases, sample types, amplification strategies, detection times and limits of detection (LOD).

Detection Method/Principle	Biomarker	Disease	Analyzed Sample Type	Signal Amplification	Target Amplification	Detection Time	LOD	Ref.
**Colorimetric Sensors**								
*microRNAs*								
DNA-functionalized AuNP aggregation	miR-4739	Breast cancer	Blood	Isothermal MNAzyme	N/A	2.5 h	7 pM	**[12]**
*Circulating tumor DNA*								
G-quadruplex/ hemin DNAzyme/ABTS	ctDNA (PIK3CA^E545K^ mutation)	Breast cancer	Serum	N/A	Triple branched HCR	>4 h	0.65 fM	**[15]**
*Exosomes*								
Graphene oxide nanozyme with peroxidase activity/TMB	Exosomes	Breast cancer	Plasma	Adenosine triphosphate-enhanced peroxidase activity	N/A	25 min	3.8 × 10^5^ particles/mL	**[14]**
DNA-functionalized AuNP aggregation	Exosomes	Leukemia	FBS	Double spherical nucleic acids	TdT reaction	1 h	45 particles/μL	**[16]**
G-quadruplex/ hemin DNAzyme/TMB	Exosomes	Leukemia	Serum	Spherical nucleic acid	HCR	>4 h	50 particles/μL	**[17]**
PDA aptasensors	Exosomes	Cancer	Serum	N/A	N/A	20 min	~3.7 × 10^4^ particles/μL	**[18]**
**Fluorescent Sensors**								
*microRNAs/piRNAs*								
Fluorescence quenching	multiple piRNAs	Cancer	Plasma	Spherical nucleic acid–AuNP conjugates	N/A	4 h	110–480 pM	**[8]**
FAM and BHQ1-labeled MBs	miR-21/piR-20365/EVs	Breast cancer	EVs/plasma	DNAzyme-enabled recycling of substrate “cleavage-binding”	N/A	30 min	3.98 and 2.69 particles/μL and 12 aM of miR-21	**[13]**
Fluorescence quenching	miR-let-7a	Cancer	Serum	Microgels conjugated with LNAs-MBs	N/A	30 min	1.3 fM	**[19]**
*Circulating tumor DNA*								
Fluorescence quenching	ctDNA	Pancreatic ductal adenocarcinoma	Serum	N/A	EXPAR	16 min	0.1% and 25 aM	**[20]**
Fluorescence quenching	ctDNA	Lung cancer	Serum/A549 cell lysates	CsPbBr_3_ nanosheets	Strand displacement	>1 h	180 fM	**[21]**
Fluorescent label	ctDNA	KRAS mutations	Cell lysates	N/A	Selective PCR	>30 min	1% mutant DNA	**[22]**
*Circulating tumor cells*								
Fluorescence quenching	CTCs	Cancer	Blood	AuNPs conjugates as three-dimensional network nanovehicle	N/A	30 min	2 cells/mL	**[23]**
*Proteins*								
Cu-Zr MOF	HER2/ER/PR/ Ki-67 proteins	Breast cancer	Serum	Magnetic adsorption separation for noise reduction/peroxidase activity of MOF	N/A	12 min	0.37–0.39 pg/mL	**[1]**
**Electrochemical Sensors**								
*microRNAs*								
Amperometry	miR-181b	Osteosarcoma	Serum	Ligase chain reaction amplification, enzyme-linked DNA MMB	N/A	45 min	6.7 aM	**[25]**
DPV	miR-9	Lung cancer	Human serum	AuNSs deposited on FTO electrodes	N/A	N/A	0.012 aM	**[26]**
DPV	miR-31	Oral squamous cell cancer	Saliva	CRISPR/Cas12a	Isothermal amplification reactions	1 h	3.5 fM	**[27]**
SWV	mir-652	Breast cancer	Serum	N/A	N/A	30 min	0.4 nM	**[28]**
SWV	miR-1246	Breast cancer	Blood	CRISPR/Cas12a, DSN, MNPs	N/A	2 h	50 aM	**[31]**
*Circulating tumor DNA*								
DPV	ctDNA	Cancer	Spiked serum	Nicking enzyme	HCR	>2 h	2.3 fM	**[29]**
Exosomes/extracellular vesicles								
DPV	Small EVs	Breast cancer	Human small EVs	Colloidal quantum dot (CQD) conjugates	N/A	N/A	19 particles/mL	**[30]**
Chronoamperometry	Exosomes	Breast	Serum	DNAzyme and enzyme-catalyzed	Ligation reaction	2 h	3.63 × 10^4^ particles/mL	**[32]**
EIS	αvβ6 integrin receptor derived from EVs	Cancer	Buffer	AuNSs deposited on paper-based carbon electrodes	N/A	1 h	0.7 × 10^3^ S-EVs/mL	**[33]**
*Proteins*								
DPV	HER 2	Breast cancer	Spiked serum	Nanodiamond/AuNP-nanohybrid-functionalized electrodes	N/A	50 min	0.29 pg/mL	**[34]**
*Circulating tumor cells*								
DPV	Mucin1/ circulating tumor cells	Lung cancer	Blood	DNA nanomachines (Y-shaped)	N/A	45 min	1 ag/mL 1 cell/mL	**[35]**
**SPR Sensors**								
*Proteins*								
Sandwich immunoassay	CSPG4 protein	Melanoma cancer	Cell culture	N/A	N/A	30 min	-	**[36]**
M13-bacteriophage decorated AuNPs	Carcinoembryonic antigen	Cancer	Serum	SPR-probe AuNPs	N/A	5 min	0.83 fM	**[37]**
Ag nanodome	Neuron-specific enolase	Small-cell lung cancer	PBST	Ag nanodome	N/A	100 min	270 pM	**[38]**
*Circulating tumor cells*								
4 different nitride layers (AlN, GaN, InN and Si_3_N_4)_	CTCs	Adrenal gland, breast, cervical, blood and skin cancer	Cancer cells/blood	Nitride layers (AlN, GaN, InN and Si_3_N_4)_	N/A	-	-	**[39]**
**SERS Sensors**								
*Circulating tumor cells*								
Mesoporous three-dimensional AuNSs	CTCs	Lung cancer	PBMCs	AuNSs	N/A	-	-	**[40]**
SERS-functionalized platform with stem cells	Metastasis-initiating stem cells	Lung cancer	Blood	N/A	N/A	-	Single-cell	**[41]**
*Metabolites*								
Raman reporter malachite green dye	Urine metabolites	Pancreatic, prostate, lung and colorectal cancer	Urine	Gold nanosponges	N/A	-	1.23 nM	**[9]**
Three-dimensional plasmonic hexaplex AuNSs	Saliva metabolites	Lung cancer	Saliva	AuNSs	N/A	-	0.13 nM 1.63 nM	**[10]**
**CRISPR-based Sensors**								
*Circulating tumor DNA*								
Release of Cy5 fluorescein	ctDNA	Lung cancer	Serum	CRISPR/Cas12a system	N/A	>4 h	5.6 fM	**[43]**
Aptamer chemiluminescent/thioflavin T system	ctDNA	-	Artificial urine/serum	Thioflavin T photocatalytic oxidase activity	N/A	20 min	86 nM	**[44]**
AuNP aggregation	cfDNA	Breast cancer	Cell lysates	CRISPR-Cas12a system	N/A	1 h	0.08 fM	**[45]**
*Exosomes*								
Aptamer chemiluminescent system	Exosomes (EpCAM- and MUC1-positive)	Breast cancer	Plasma	CRISPR/Cas12a system	N/A	-	1.45 × 10^2^ and 3.73 × 10^2^ particles/μL	**[47]**
***RCA-based Sensors***								
Fluorescent intercalating dye	Circular RNA circHIPK3	Breast cancer	Cancer cells	Specific primer	RT-HRCA	2 h	6.6 aM	**[48]**
*Exosomes/extracellular vesicles*								
Dimerized guanine quadruplex/thioflavin T system	Exosomes	Breast cancer	Serum	Immunomagnetic separation for preconcentration	ERCA	>3 h	2.4 × 10^2^ particles/mL	**[49]**
Calcein fluorescence	Exosomes	Lung cancer	Blood	Coordination of Cu^2+^ with dsDNA	HCR	>2 h	100 particles/mL	**[50]**
Fluorescent MOFs	EVs	Leukemia	Serum	N/A	RCA	>2 h	2.2 × 10^4^ particles/μL	**[51]**
Fluorescence Quenching	EVs	Breast cancer	Plasma	CRISPR	HCR	2 h	3.3 × 10^2^ particles/μL	**[42]**
***Lateral flow assays (LFAs)***								
*microRNAs*								
FRET- AuNPs conjugates quenchers	miR-21	Periodontitis, lung cancer	Saliva and Serum	Ag shell of AuNPs	N/A	20 min	1 fM–2 nM	**[53]**
AuNPS/DNA tetrahedral probes	microRNA-150–5p	Diabetic nephropathy	HK-2 cells	CHA	CHA	65 min	58.90 fM	**[54]**
SERS	microRNA-133a	Acute myocardial infarction	Spiked human serum	CRISPR/Cas12a system	CHA	>2 h	0.32 fM	**[55]**
Visual detection	miR-21 and miR-let-7a	Bladder cancer	Spiked human urine	AuNPs	RT-PCR	>2 h	10^2^ miRNA copies	**[56]**
*Exosomes*								
Fluorescent aptamer-microspheres	Exosomes	Breast cancer	Serum	N/A	N/A	85 min	2.5 × 10^3^ particles/mL	**[52]**
**Other sensors**								
Smartphone-enabled visualization on microfluidic platform	Nematode movement	Breast cancer metastasis	Urine	N/A	N/A	65 min	-	**[57]**

N/A: not available; AuNPs: gold nanoparticles; MNAzyme: multicomponent nucleic acid enzymes; HCR: hybridization chain reaction; TdT: terminal deoxynucleotidyl transferase; TMB: tetramethylbenzidine; PDA: polydiacetylene; LNAs: Locked nucleic acids; MBs: Molecular beacons; piRNAs: PIWI-interacting RNAs; EXPAR: exponential amplification reaction; EVs: extracellular vesicles; ERCA: exponential rolling circle amplification; MOF: metal–organic framework; AuNSs: gold nanostructures; CHA: catalytic hairpin assembly; FTO: fluorine-doped tin oxide; DSN: duplex specific nuclease; MMB: magnetic microbead; Ty-AuNPs: tyramine-modified gold nanoparticles; MNPs: magnetic nanoparticles; DPV: differential pulsed voltammetry; HER2: human epidermal growth factor receptor 2; SWV: square wave voltammetry; EIS: electrochemical impedance spectroscopy; RCA: rolling circle amplification; RT-HRCA: reverse transcription–hyperbranched rolling circle amplification; FRET: fluorescence resonance energy transfer; PCR: polymerase chain reaction.

## Data Availability

Data sharing is not available.
